# Study on Regulation of Low Density Lipoprotein Cholesterol Metabolism using PCSK9 Gene Silencing: A computational Approach

**DOI:** 10.6026/97320630014248

**Published:** 2018-05-31

**Authors:** Bhooma Vijayaraghavan, Kavitha Danabal, Giri Padmanabhan, Kumaresan Ramanathan

**Affiliations:** 1 Kidney Care, C50, 10TH B Cross, East Thillai Nagar, Tiruchirappalli-620 018, India; 2 Department of Botany & Microbiology, AVVM Sri Puspam College (Autonomous), Poondi, Thanjavur, India; 3 Department of Medical Biochemistry, Division of Biomedical Sciences, School of Medicine, College of Health Sciences, Mekelle University (Ayder Campus), Mekelle, Ethiopia

**Keywords:** PCSK9 mRNA, siRNA, LDL, free energy of hybridization, knock down

## Abstract

Combating and preventing abnormality in lipid metabolism becomes a pivotal criterion for research. Proprotein convertase subtilisin/kexin type 9 (PCSK9) is a circulating protein; it promotes the degradation of low-density lipoprotein receptors (LDL-R) and hence increases LDL-C levels. Silencing the gene PCSK9 at post-transcriptional level with the help of small interfering Ribo nucleic acid (siRNA) gives a new insight and a novel therapeutic way to regulate LDL-C metabolism. Designing and selecting an efficient siRNA for silencing PCSK9 at post transcriptional level through computational approach. We have designed three siRNAs to silence each mRNA of PCSK9 through computational analysis using software Invivogen. Their minimum free energy of hybridization along with their secondary structure was obtained using bioinformatics tool BIBISERV2-RNAHYBRID. Further factors like GC content, structural linearity and h-b index of mRNA-siRNA complex was calculated to assess their knockdown efficiency. The minimum free energy of hybridization of the three designed siRNA1, siRNA2 and siRNA3 for target mRNA is as follows -27.1kcal/mol, -25.7kcal/mol and - 28.8 kcal/mol. siRNA1 having the least minimum free energy of hybridization i.e. -27.1 kcal/mol are predicted to be the most efficient towards the PCSK9 gene silencing.

## Background

Amid the previous couple of years, the proprotein convertase subtilisinkexin 9 (PCSK9) field has been scorching, powered by the acknowledgment that PCSK9 is a key player in plasma cholesterol digestion system and by a trust, shared by researchers in the educated community and industry alike, that PCSK9 is an objective for treating hypercholesterolemia. PCSK9 directs the levels of the LDL receptor [1, 2, 3], which is a plasma membrane glycoprotein that expels cholesterol rich LDL particles from the plasma [4, 5]. PCSK9 is an individual from the mammalian serine proprotein convertase family that commonly capacities in the proteolytic handling and development of secretory proteins [6, 7]. PCSK9 was the principal relative to be embroiled in an overwhelmingly acquired type of hypercholesterolemia [8]. The human hereditary and quality expression disclosures started enthusiasm for characterizing PCSK9 capacity. Mice trial immediately settled a connection amongst PCSK9 and levels of LDL receptors in the liver. Adenoviral over expression of PCSK9 in mice brought about higher LDL cholesterol levels and lower levels of LDL receptors, without changing LDL receptor mRNA levels [1, 3, 9]. Thus, PCSK9 transgenic mice displayed expanded LDL cholesterol levels, and LDL receptors in the liver were for all intents and purposes killed [10]. Parabiosis concentrates on set up that the PCSK9 from a transgenic mouse decreased LDL receptor levels in the liver of a matched non-transgenic mouse (10). Along these lines, flowing PCSK9 brings down LDL receptor levels. Then again, thumping out PCSK9 expanded hepatic LDL receptors and lessened plasma cholesterol levels [11].

A few methodologies for repressing PCSK9 capacity are hypothetically plausible. Since autocatalytic cleavage is required for the development of PCSK9, a little particle inhibitor of autocatalysis may be helpful [3], gave that it was particular to PCSK9 handling and did not prompt a poisonous gathering of misfolded PCSK9. Little atoms that square the PCSK9 LDL receptor co-operations would likely be strong, albeit outlining inhibitors of protein connections is a difficult request. Acceptance of PCSK9 as an appealing remedial focus for the treatment of hypercholesterolemia has gotten to be inevitable. Small interfering RNAs (siRNAs) have been as of late recognized as vital controllers of qualities required in cholesterol homeostasis and potential novel helpful focuses for hypercholesterolemia. Small interfering RNAs (siRNA) are small (~22 nucleotide) single-stranded RNA particles that manage quality expression dominatingly at the posttranscriptional level. siRNAs have been as of late recognized as vital controllers of qualities required in cholesterol homeostasis and potential novel helpful focuses for hypercholesterolemia [12]. Subsequently the present study focused on outlining and assessing the adequacy siRNA to hush the PCSK9 gene at the small-scale RNA level using computational methodology.

## Methodology

### Sequence Retrieval

The coding sequence of PCSK9 mRNA was collected from NCBI Database (http://www.ncbi.nlm.nih.gov/). The Accession number of target PCSK9 mRNA is NM_ 174936.3. The sequence was retrieved in FASTA format and used as a target gene for designing functional siRNA molecules.

### Target Recognition and Potential siRNA design

Target identification was carried out in PCSK9 mRNA sequence using online Bioinformatic software Invivogen siRNA wizard (http://www.invivogen.com/sirnawizard/design.php). siRNA target site was designed using standard human database with 21 nucleotide motif size. The tool excludes siRNA with palindrome sequence, high GC content and performs blast search to reduce off target similarity.

### Calculation of GC content

To assess the correlation between GC content and siRNA accessibility, GC content of all the designed siRNA was calculated using online GC calculator (http://www.endmemo.com/bio/gc.php). siRNA molecules with low GC (40% to 55%) were chosen to analyse knockdown efficiency: The GC content was calculated using the below formula: (Number of G nucleotide + Number of C nucleotide) in the sequence X 100 = GC content% / Total number of nucleotide in the sequence

### Calculation of RNA-RNA interaction through thermodynamics

The designed siRNA was screened for effective knockdown analysis using thermodynamic interface. Thermodynamic RNARNA interaction between the siRNA (target strand) and target PCSK9 (mRNA) sequence was carried out using online Bioinformatic software BIBISERV2-RNAHYBRID [13]. The tool functions as an extension of Dynamic programming algorithm to compute the hybridization energy, their Structure and basepairing form of two RNA sequences. It also utilizes parameters like target source (human) and hits per target (one) to compute probabilities of base pairing and realistic interaction energies of mRNA-siRNA duplex. Hybridization structure was used to evaluate various factors like structural linearity and h-b index.

## Results

**Figure 1:**
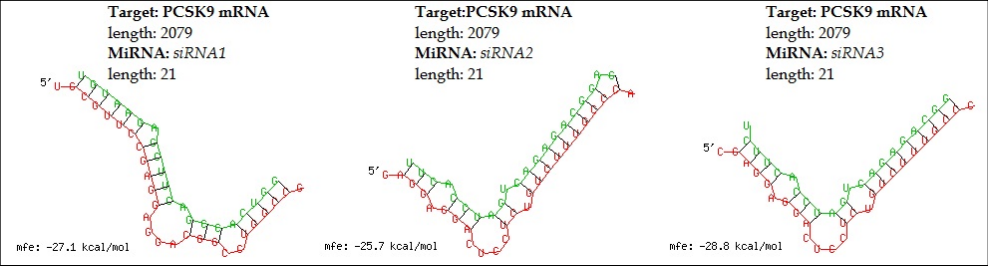
Predicted secondary structure showing minimum free energy of hybridization between target PCSK9 mRNA and a) siRNA1 b) siRNA2 and c) siRNA3

This study was undertaken to evaluate the designed siRNA for their effective knock down analysis. Invivogen siRNA wizard tool predicted three siRNA from the target sequence without a specific database search (Table 1). Manually designed siRNA using complementary base pair rule followed by uracil substitution from the recognized target sequence. siRNA 1 has a target sequence(GGTCACCGACTTCGAGAATGT) identified at 603position from which the designed siRNA guide strand sequence is CCAGUGGCUGAAGCUCUUACA. siRNA 2 has a target sequence(GAGGCAGAGACTGATCCACTT) identified at 1233position from which the designed siRNA guide strand sequence is CUCCGUCUCUGACUAGGUGAA. siRNA 3 has a target sequence(GGCAGAGACTGATCCACTTCT) identified at 1235 position from which the designed siRNA guide strand sequence is CCGUCUCUGACUAGGUGAAGA. From the predicted three-siRNA sequence, only one sequence (CCAGUGGCUGAAGCUCUUACA) has been identified sequence specific for human database using BLAST search. According to Blast specificity analysis result, siRNA 1 was found to be species specific for human and mouse. siRNA 2 was found to be species specific for Mouse and rat. However siRNA 3 was not species specific for Human, Mouse and Rat. The knockdown efficiency of siRNA was analyzed based on thermodynamic RNA- RNA interaction using the hybridization energy between the target PCSK9 mRNA and guide strand of siRNA for effective RNAi activity. The minimum free energy of hybridization of the designed three siRNA's are as follows: -27.1kcal/mol, - 25.7kcal/mol and -28.8 kcal/mol respectively. Since the siRNA3 did not show species specificity for human, mouse and rat database, it was neglected. The best-fitted siRNA was predicted between siRNA1 and siRNA2. The hybridization structures in Figure 1 represents PCSK9 mRNA (red) with siRNA (green) hybrid and were analyzed for parameters like structural linearity, h-b index and GC content of the siRNA. The corresponding GC Content was found is same for all the three-designed siRNA that is 52.38%.

## Discussion

This study was conducted with target sequence retrieval and analysis. The target PCSK9 mRNA having sequence length of 3731 base pairs was retrieved from NCBI in FASTA format. This sequence was utilized as a template for designing target specific functional siRNA by Invivogen siRNA wizard software. The tool identifies sequence specific siRNA with low targets by employing certain processes such as Thermodynamic, GC content analysis, BLAST search, secondary structure avoidance, Termination signal and immunostimulatory motif exclusion (http://www.invivogen.com/sirna-wizard).

siRNA Wizard tool predicted three potential target sites on PCSK9 mRNA for siRNA binding.The tool predicts three desired target sequences present in the target mRNA sequence. Desired siRNA sequence was designed using the target sequences. BLAST search was undertaken to eliminate the non-specific siRNA using seed human database. Certain nucleotide sequences such as GCCGGC are responsible for stimulating immunological response [14]. Hence immunostimulatory motif exclusion was performed in designing siRNA.

Efficiency of siRNA in RNAi activity was assessed by calculating the RNA-RNA interaction through thermodynamics. The interaction between the siRNA (guide strand) and mRNA (PCSK9) was utilized to predict the gene silencing efficiency of siRNA. Minimum free energy of hybridization was calculated between the siRNA and target PCSK9 mRNA hybrid. Secondary structures of RNA play a vital role in determining the efficiency of post-translational gene silencing [15, 16]. Bioinformtic software BIBISERV2-RNAHYBRIDfrom Bielefeld Bioinformatics Service was used for the prediction of hybridization structure and energy [13]. The software follows dynamic programming algorithm for prediction of secondary structure. The hybridized structure of 6 designed siRNA with target PCSK9 mRNA was shown in Figure 1.

The hybridized structure was evaluated for various parameters like h-b index, structural linearity of siRNA-mRNA complex and GC content of siRNA. The linearity was analyzed using the h-b index and lowest degree of nucleotide presence in loop region. Hb index is inversely proportional to linearity and RNAi activity. Negative correlation was established between RNi activity and GC content of siRNA in prior studies [17, 18]. The GC content of the designed siRNA was found to be in the range between 40% and 55%. Even the best-fitted siRNA was found to be within the range of low GC content.

siRNA 1 having the least minimum free energy of hybridization i.e.-27.1 kcal/mol, it is predicted to be the most efficient towards the PCSK9 gene silencing. Moreover siRNA 1 is found to be species specific for both human and mouse. An animal study (mouse) will help in estimating efficacy of siRNA and their correlation with human in analyzing the efficiency of RNAi activity. siRNAs have been as of late recognized as vital controllers of qualities required in cholesterol homeostasis and potential novel helpful focuses for hypercholesterolemia [12]. We also deduced that the binding efficiency varies with factors like linearity of mRNA, h-b index, low GC content of siRNA. Of all the factors h-b index of mRNA plays the most influential role in establishing a better knock down efficiency. If the mRNA has low h-b index, it tends to be more linear and hence binding of siRNA is more efficient.

## Conclusion

Conventional therapeutic strategy has failed to overcome disorder, which ultimately necessitates the establishment of alternative strategy. Hence molecular therapeutics has been elevated to show its therapeutic approach at genomic level. siRNA molecules proved its effectiveness in curing many incurable disease. Hence, we have evaluated siRNA efficiency for effective RNAi activity. Further in vivo studies have to be carried out to confirm the knock down efficiency.

## Conflicts of Interest

None declared

## Figures and Tables

**Table 1:** Predicted target site and designed sense strand of siRNA for target PCSK9 mRNA.

Target site of PCSK9 mRNA	Design of sense siRNA	Location in PCSK9 mRNA	GC content%
GGTCACCGACTTCGAGAATGT	CCAGTGGCTGAAGCTCTTACA	603	52.38
GAGGCAGAGACTGATCCACTT	CTCCGTCTCTGACTAGGTGAA	1233	52.38
GGCAGAGACTGATCCACTTCT	CCGTCTCTGACTAGGTGAAGA	1235	52.38
